# Experimental Investigation of Strain Rate Influence on Anisotropy of Uniaxial Tensile Mechanical Properties of CuFe2P Alloy Sheet

**DOI:** 10.3390/ma17133135

**Published:** 2024-06-26

**Authors:** Ante Bubalo, Zdenko Tonković, Lovre Krstulović-Opara, Vedrana Cvitanić

**Affiliations:** 1 Yazaki Europe Limited, Slavonska 26/6, HR-10000 Zagreb, Croatia; 2 Faculty of Mechanical Engineering and Naval Architecture, University of Zagreb, Ivana Lučića 5, HR-10000 Zagreb, Croatia; zdenko.tonkovic@fsb.unizg.hr; 3 Faculty of Electrical Engineering, Mechanical Engineering and Naval Architecture, University of Split, R. Boškovića 32, HR-21000 Split, Croatia; lovre.krstulovic-opara@fesb.hr (L.K.-O.); vedrana.cvitanic@fesb.hr (V.C.)

**Keywords:** copper alloy sheets, strain rate, material anisotropy, thermography, DIC

## Abstract

Wire crimping, a process commonly used in the automotive industry, is a solderless method for establishing electrical and mechanical connections between wire strands and terminals. The complexity of predicting the final shape of a crimped terminal and the imperative to minimize production costs indicate the use of advanced numerical methods. Such an approach requires a reliable phenomenological elasto-plastic constitutive model in which material behavior during the forming process is described. Copper alloy sheets, known for their ductility and strength, are commonly selected as terminal materials. Generally, sheet metals exhibit significant anisotropy in mechanical properties, and this phenomenon has not been sufficiently investigated experimentally for copper alloy sheets. Furthermore, the wire crimping process is conducted at higher velocities; therefore, the influence of the strain rate on the terminal material behavior has to be known. In this paper, the influence of the strain rate on the anisotropic elasto-plastic behavior of the copper alloy sheet CuFe2P is experimentally investigated. Tensile tests with strain rates of 0.0002 s^−1^, 0.2 s^−1^, 1 s^−1^, and 5.65 s^−1^ were conducted on sheet specimens with orientations of 0°, 45°, and 90° to the rolling direction. The influence of the strain rate on the orientation dependences of the stress–strain curve, elastic modulus, tensile strength, elongation, and Lankford coefficient was determined. Furthermore, the breaking angle at fracture and the inelastic heat fraction were determined for each considered specimen orientation. The considered experimental data were obtained by capturing the loading process using infrared thermography and digital image correlation techniques.

## 1. Introduction

**Figure 1:**
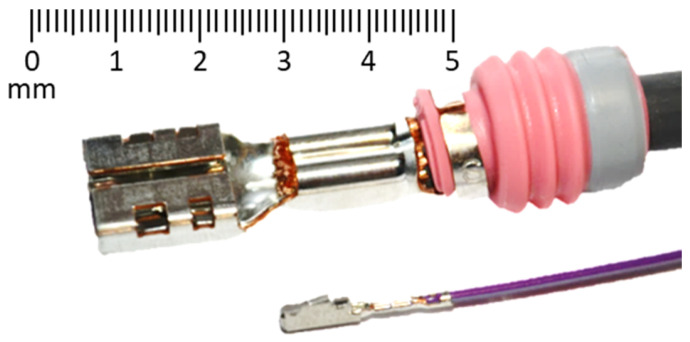
Terminal strip with crimped terminal.

The wire crimping process is widely used in the automotive industry. It is a metal-forming process where the terminal is drastically deformed into a new shape (Figure 1). This process is conducted at higher velocities, wherein wire crimping tools may travel with a velocity of up to 0.5 m/s [1]. The quality of a crimped connection is critical, as it must withstand various mechanical loads and environmental conditions without compromising its integrity [2,3]. Due to the minimization of all parts in the automotive industry and new demands on reliability and safety, together with the applications of new materials and new production technologies, further studies are required. In this sense, one possibility is the use of advanced numerical models. The final results of numerical simulations will significantly depend on how accurately the constitutive model is defined [4]. Copper and its alloys are widely used in electrical applications due to their excellent electrical conductivity and ductility. Copper alloy sheets, such as CuFe2P, are commonly used for manufacturing terminals due to their improved tensile strength and durability. However, to accurately predict the behavior of copper alloy sheets during the crimping process [5], detailed knowledge of their material properties is essential. The behavior of material under crimping conditions is complex and is influenced by the rate at which the strain is applied, known as the strain rate. Unfortunately, the stress–strain relationships for copper alloy sheets, particularly at the higher strain rates typical for the crimping process, are not well documented [6].

Copper alloy sheets that are produced with the rolling process may have severe anisotropy due to changes in grain morphology [7]. After rolling, the grain size is reduced and stretched in a rolling direction [8]. This creates a specific texture, known as a rolling texture. The grains tend to become elongated along the rolling direction (RD), widened in the transverse direction (TD), and flattened in the normal direction (ND), often described as ‘pancake-shaped’ [9]. Rolling texture causes anisotropy in tensile mechanical properties such as yield stress, strength, and ductility, i.e., these properties might vary with the direction of tensile loading relative to the rolling direction. In other words, the presence of a rolling texture can lead to directional dependences on these properties that must be considered in the design and manufacturing of copper components.

In metal sheets, the fracture behavior is often not readily observable. This phase is critical, as material degradation can lead to various forms of necking and failure. Consequently, researchers have been drawn to investigate the influence of post-necking behavior. Necking is a localized reduction in the specimen’s cross-sectional area that occurs during uniaxial tension. This phenomenon typically arises after the material has reached its ultimate tensile strength (UTS) and is undergoing plastic deformation. The start of necking marks the transition from uniform to non-uniform deformation and is a precursor to fracture. Here, the angle between the loading direction and the fracture surface plane is defined as the fracture angle. This angle is a critical parameter that reflects the material’s failure mode under tensile loading [10]. Experimental and numerical modeling results show that the fracture angle of the tensile specimen is highly dependent on the ratio of its thickness and width, known as the aspect ratio (AR). Larger AR values lead to fracture angles close to 55°, while lower AR values result in fracture angles approaching 90° [11].

To characterize the anisotropy of sheet material in plastic flow, the Lankford coefficient (often denoted as the *r*-value) is commonly utilized. This coefficient is defined as the ratio of increments of transverse and thickness true plastic strains obtained in a tensile test of the sheet specimen. Based on the incompressibility condition, the Lankford coefficient can be calculated using plastic strain increments in the longitudinal and transverse directions, i.e., by the slope of the selected curve fit for the transverse versus longitudinal true plastic strain diagram obtained in a tensile test. By a common approach, a linear fit with an intercept at the origin is applied to the whole strain diagram, and thus the Lankford coefficient is considered a constant value. Several recent studies indicate that the Lankford coefficient should be considered as a value that is altered by the ongoing deformation process in tensile testing [12,13,14,15,16]. In these studies, two approaches can be found. In the first approach, the transverse versus longitudinal true plastic strain diagram is divided into several intervals with equal longitudinal strain sizes. Then, incremental Lankford coefficients are calculated by applying linear fits with a free intercept for each strain interval. In the second approach, a single non-linear fit is applied to the whole strain diagram, and the Lankford coefficient is considered an instantaneous value defined by the fit’s slope corresponding to the considered longitudinal strain. The values of the Lankford coefficient obtained in the tensile tests of specimens with different alignments to the rolling direction are utilized in calibrating phenomenological anisotropic plasticity models for sheet metals. Anisotropic models that take into account anisotropy evolution during the deformation process, i.e., utilize incremental or instantaneous *r*-values in the calibration procedure, might result in improved predictions of complex sheet metal-forming processes [12,15,16].

Despite the fact that copper alloys are widely used in the automotive industry, where a lot of optimization is required, to our knowledge, no study has considered copper alloy sheet anisotropy. As indicated above, several studies report the alternation of the Lankford coefficient during tensile testing for various sheet metals. Moreover, only one study discusses the Lankford coefficient being influenced by strain rate for specific steel and aluminum alloy sheets [17]. Experimental research on dynamic strain aging in an AlMg alloy at different strain rates and temperatures provides valuable insights into how these factors influence material behavior [18]. Furthermore, a multiscale experimental study on TWIP steel investigates anisotropy and strain rate effects on failure behavior, emphasizing the significant impact of these factors on material performance [19]. Research on the high-strain-rate behavior of an AA6016 alloy sheet in T4 and T6 tempers for sheet metal-forming processes highlights the impact of strain rate on ultimate tensile strength and elongation for the considered material [20].

Conventional methods applied in tensile tests, where strain is measured between two points on the specimen, e.g., extensometers, strain gauges, etc., will provide reliable results regarding the elastic modulus but not reliable results for yield point and local strain distribution [21,22]. The main issue that arises when strain is measured between two points is that the result determines a global strain value that may not accurately reflect the severity of the strain during metal-forming processes. For wire crimping and most metal-forming processes, it is mandatory to know the upper strain limit, and it needs to be measured locally as there may be differences due to necking [23].

In particular, to our knowledge, no study has considered the experimental investigation of the anisotropic material behavior of CuFe2P copper alloy sheets at different strain rates. Only one article evaluates the strain rate dependence of tensile strength and ductility in nano- and ultrafine-grained coppers [24]. For this purpose, in this study, a combination of two experimental methods is utilized. The first method involves using the two-dimensional (2D) optical measurement system ARAMIS 4 M [25]. In combination with the digital image correlation (DIC) method, displacement field and surface strain distribution during a tensile test can be obtained [26]. The second measuring method is based on the use of infrared (IR) thermography, where a fast-cooled InSb middle-wave thermal camera measures temperature changes on material surfaces [27,28,29]. When a material is stressed up to the yield point, the camera detects a small temperature drop, but after the material passes the yield point and starts to deform plastically, the temperature rises. With this method, it is possible to precisely detect yield points and yield propagation directions. As IR thermography is a non-contact method that measures radiance, the results and accuracy will depend on emissivity, ambient temperature, focus, and other thermographic parameters. The accuracy of IR cameras is ±2%, and for the specific model used in this research, ±1% or ±1 °C [30].

Non-contact methods like DIC and IR will provide more accurate results as they do not influence specimen behavior. Both methods are full-field methods showing surface strain and temperature distribution, contrary to conventional methods that can provide only local, pointwise results. A conventional extensometer or strain gauge may locally increase stiffness and change stress distribution. Also, in combination with a temperature probe, heat flow may influence the temperature reading. Furthermore, the two methods considered have some limitations that are opposite to each other. The DIC method is more appropriate for lower test velocities as exposure time can be longer and blurred images will be eliminated. The optical measurement system Aramis 4 M is limited by the frame rate, which can go from 1 Hz up to 480 Hz. For higher frame rates, an adequate light source needs to be used, as camera exposure time would be lower. For infrared IR thermography, it is desirable to have higher velocities, as it will reduce heat dissipation and the process will be closer to an adiabatic process showing a clear reading of temperature distribution. A quasi-static test cannot be measured with the IR method, as heat will dissipate within the material between the elastic zones and the zones where yielding occurs. The thermal camera used in this work is based on a cooled InSb photonic detector with a frame rate of up to 700 Hz, which is an adequate acquisition rate for this particular investigation.

Several papers deal with the determination of the fraction of plastic work that is converted into heat during the plastic deformation of metals (denoted as *β*). *β* is also referred to as the inelastic heat fraction (IHF) or Taylor–Quinney coefficient. In many finite element codes, *β* is assumed to be constant, typically around 0.9 for metals [31]. Paper [32] proposed a new method to identify *β* during a quasi-static tensile test using an inverse Finite Element Update approach with simultaneous digital image correlation (DIC) and infrared (IR) thermal measurements. According to this approach, its value is not constant and can depend on strain, strain rate, and temperature. Accurate determination of *β* is essential for predicting temperature variations during deformation and understanding the material’s response to mechanical loads [33].

In this work, the anisotropic elasto-plastic behavior of the copper alloy CuFe2P sheet in tensile testing at different strain rates was investigated using a combination of infrared thermography and the DIC method. The primary objective of the conducted experiments was to provide valuable results that could be used for further numerical investigations. Here, material anisotropy denotes the directional dependence of a material’s properties, meaning that the behavior of the material can vary based on the direction in which it is tested. This phenomenon is particularly important in metal sheets, where a manufacturing process can significantly influence the material’s anisotropic characteristics.

This paper is organized as follows: Section 2 contains a description of the experimental investigations. Here, the uniaxial tensile tests are performed under different strain rates on sheet specimens with different alignments to the rolling direction. Therein, complete procedures, equipment, and measurement parameters are described in detail. Next, in Section 3, the experimental results are presented together with a discussion about the influence of strain rate on the anisotropic behavior of the considered material. The results obtained by using the DIC and IR methods are demonstrated and compared. Finally, concluding remarks are given in the last section.

## 2. Experimental Investigation

In the wire crimping process, there are at least two different metal materials involved. Alloyed copper with improved tensile strength is used for terminals, and pure copper is used for wire strands. In this study, the mechanical behavior of the copper alloy sheet CuFe2P was thoroughly investigated to understand its performance under various loading conditions. The chemical composition of the CuFe2P alloy is given in weight percent (wt%) in Table 1, according to the supplier datasheet received. The material used in this study, CuFe2P, was sourced from KME Germany GmbH as A4-sized sheet metal plates.

**Figure 2:**
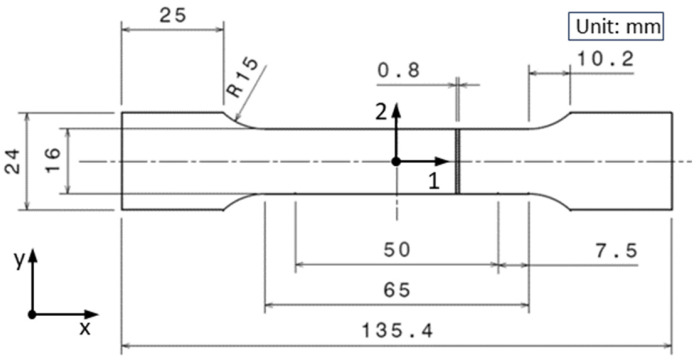
Tensile specimen geometry.

Tensile test specimens were made of CuFe2P sheet with a thickness of 0.8 mm; specimen size was 135.4 mm × 24 mm × 0.8 mm (Figure 2). Each specimen was cut using a wire EDM (Electrical Discharge Machining), which enables high-precision cuts following a defined contour. To characterize material anisotropy in mechanical properties, tensile tests were conducted on specimens whose longitudinal axes 1 were oriented at 0°, 45°, and 90° relative to the rolling direction x.

Tensile tests were performed at four different strain rates, according to Table 2. This range of velocities was chosen to simulate the various strain rates that the material might experience during different stages of the crimping process, from slow deformation to rapid loading. For the evaluation of the mechanical behavior of the tested specimens, a standard uniaxial tensile test was performed. This test method is widely recognized for its ability to provide fundamental material properties such as yield stress, ultimate tensile strength, and elongation at break, which are essential for the design and analysis of crimped connections.

**Figure 3:**
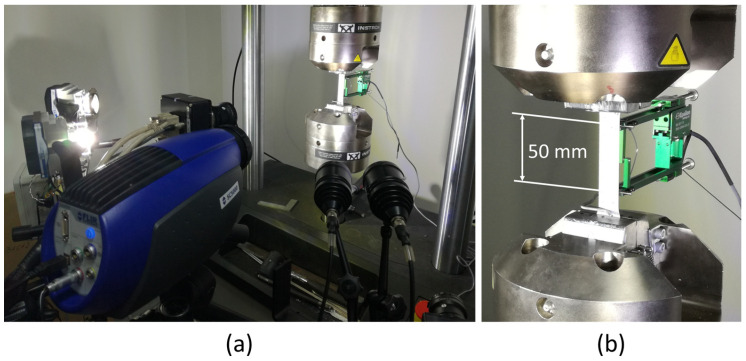
(**a**) Experimental setup with optical and IR cameras; (**b**) tensile specimen with clip-on extensometer.

During loading, specimen surface temperature and strain were acquired by a cooled middle-wave IR camera and the ARAMIS 4 M system. For additional confirmation of the DIC method, the clip-on Epsilon longitudinal extensometer was used (Figure 3). The ARAMIS system was synchronized with a thermographic camera in order to find a correlation between strain and temperature change. Due to DIC method requirements, test samples were painted with a stochastic pattern, i.e., black dots on a white background. Here, it is important to mention that the paint of both colors must be of the same origin, meaning they must show the same emissivity in the infrared spectrum. Based on these contrasting dots, ARAMIS 4 M, whose algorithm is based on the digital image correlation method, can calculate displacements of the dots during tensile test load. The influence of DIC pattern on the results is described in works [34,35]. The optical system was calibrated before the measurement with an appropriate measuring volume. The initial image, captured by the optical system, was at the unloaded stage and used as a reference image. Thermal emissivity coefficient for the used pattern was 0.85. Thermal emissivity is evaluated by a standard procedure where the specimen is heated to a uniform temperature, and emissivity is evaluated by comparing it with a part of the surface covered with a layer of materials with known emissivity. This calibration process ensured that the temperature readings from the IR camera were reliable and accurate.

**Figure 4:**
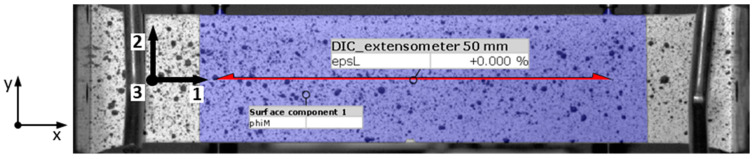
Location of elongation measurement points for DIC method at the same position where extensometer is clipped on. Specimen orientation is defined with axis 1 representing the longitudinal direction, axis 2 the width direction, and axis 3 the thickness direction.

For the estimation of the elastic modulus, digital image correlation (DIC) was used. To have DIC results comparable to those of a clip-on extensometer, elongation was measured at the same points where the extensometer was clipped, as shown in Figure 4. The initial distance between these two measured points was 50 mm. Specimen orientation is defined with axis 1 representing the longitudinal direction, axis 2 the width, and axis 3 the thickness, as indicated in Figure 2. Within the ARAMIS software, all images were discretized using square facets of size 20 × 20 pixels with an overlap of 20 × 20%. Furthermore, the DIC sampling rate enabled the measurement of the *r*-value at each increment of strain, capturing the evolution of anisotropy in plastic flow throughout the deformation process.

To ensure the acquisition of high-quality images during high-load velocity experiments, where short exposure times are necessitated by elevated frame rates, an external lighting system was implemented. This system comprised two 50 W LED air-cooled light sources, each powered by a stabilized direct current (DC) power supply to maintain consistent illumination levels and eliminate flickering. To prevent the issue of light reflection, which can obscure critical details in the recorded images, a lens equipped with a polarization filter was mounted directly onto the LED chips. The polarization filter’s ability to reduce glare and unwanted reflections enhanced the contrast and sharpness of the images, which led to a more accurate analysis of the specimen’s surface.

## 3. Experimental Results

In this study, tensile tests were performed on three samples for each tested orientation (0°, 45°, and 90° relative to the rolling direction) and for each considered strain rate value (0.0002 s^−1^, 0.2 s^−1^, 1 s^−1^, and 5.65 s^−1^). Therefore, altogether, 36 tensile tests were performed.

**Figure 5:**
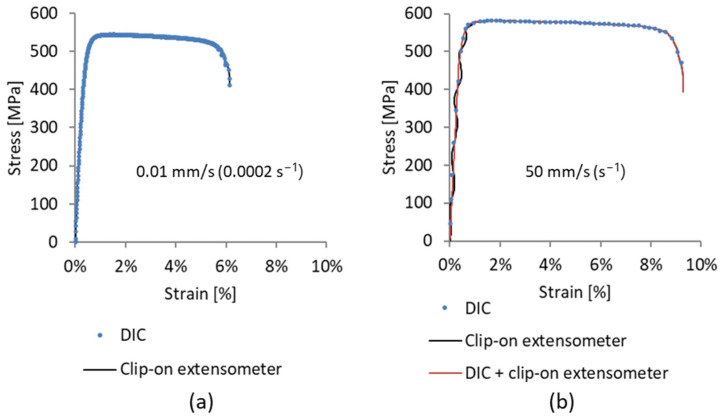
Comparison of engineering stress–strain curves for selected specimens with orientation of 0°, wherein strain is measured with the extensometer and DIC method at a strain rate of (**a**) 0.0002 s^−1^ (0.01 mm/s) and (**b**) 1 s^−1^ (50 mm/s).

The first evaluation of the results is related to the data obtained from the tensile test machine and an extensometer. An extensometer is a conventional tool used for evaluating strain and determining the elastic modulus. Due to the Aramis 4 M recording frame-rate limitation, a decrease in the number of recorded images can be observed as the strain rate increases, as shown in Figure 5. It was found that an extensometer is not an appropriate measuring tool when thin specimens are loaded at higher velocities. Starting at a velocity of 50 mm/s (1 s^−1^) and above, the wave form of the stress–strain curve in the elastic region can be seen. Only for tests performed at lower velocities (0.01 mm/s (0.0002 s^−1^) and 10 mm/s (0.2 s^−1^)), the waveform is not present, and the elastic modulus can be determined by using an extensometer only. The point is that, due to specimen thickness, extensometer readings are inaccurate for higher velocities. When a specimen is pulled at a higher velocity, the thin specimen starts to vibrate, and with the increase in tensile test velocity, the vibrations become more pronounced. After the material starts to deform plastically, the vibrations start to calm down, as shown in Figure 5. For higher velocities, a combination of a clip-on extensometer and DIC was used for the reconstruction of the stress–strain curve. As the DIC method shows accurate results during elastic and plastic deformation but has limitations in the number of recorded images, the use of a clip-on extensometer increased the number of usable points for the newly generated stress–strain curve.

### 3.1. Stress–Strain Curves

**Figure 6:**
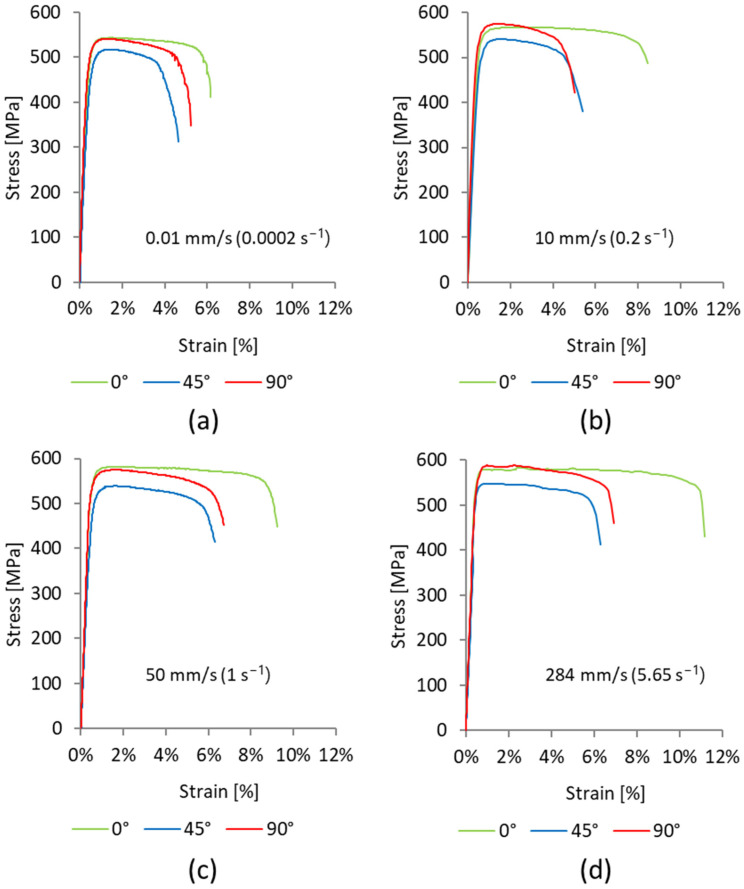
Comparison of engineering stress–strain curves calculated using DIC data for selected specimens with orientations of 0°, 45°, and 90° at strain rates (**a**) 0.0002 s^−1^, (**b**) 0.2 s^−1^, (**c**) 1 s^−1^, and (**d**) 5.65 s^−1^.

Based on the experimental data, engineering stress–strain curves for selected specimens with different alignments to the rolling direction at different strain rates were recorded and are presented in Figure 6. It can be observed that the considered material shows pronounced anisotropy in the stress–strain curve. Furthermore, strain rate significantly influences material response. For each considered strain rate, strength and elongation have the greatest values for an orientation of 0°, while the lowest values are obtained for an orientation of 45°. With the increase in strain rate, strength and elongation increased for all considered orientations.

**Figure 7:**
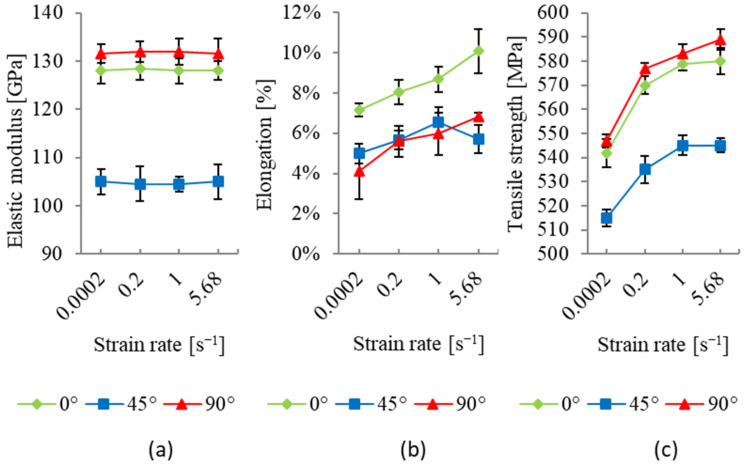
(**a**) Elastic modulus, (**b**) elongation, and (**c**) tensile strength calculated using DIC data for specimens with orientations of 0°, 45°, and 90° at different strain rates.

The experimental results shown in Figure 7 present the impact of strain rate on the mechanical properties of specimens oriented at 0°, 45°, and 90° with respect to the rolling direction. The presented results are averaged data obtained from three samples for each considered orientation, while the depicted vertical bars denote the lowest and highest measured values. The mechanical properties considered are the elastic modulus, the tensile strength, and the elongation. It can be noticed that the elastic modulus is not influenced by the strain rate but is highly dependent on the specimen’s orientation angle. When considering the reference specimen aligned with the rolling direction (0° orientation), it is evident that the one with an orientation of 90° exhibits a slightly higher elastic modulus, while the one with an orientation of 45° shows a pronounced reduction in the elastic modulus. In terms of tensile strength, a similar orientation-dependent trend is observed. Here, the specimen with an orientation of 90° has the highest tensile strength value, while the specimen with an orientation of 45° has the lowest. For all orientations, the tensile strength increases with the strain rate. The initial elongation trends diverge slightly from the patterns seen for the elastic modulus and the tensile strength. The reference specimen (0° orientation) possesses the greatest elongation, while the specimen with an orientation of 90° has the lowest. However, as the strain rate intensifies, there is an increase in elongation for specimens with orientations of 0° and 90°, while the distribution for specimens with an orientation of 45° shows one extreme for the considered strain rate range. The increase in elongation is the most pronounced for an orientation of 0°.

### 3.2. Utilization of Infrared Thermography

**Figure 8:**
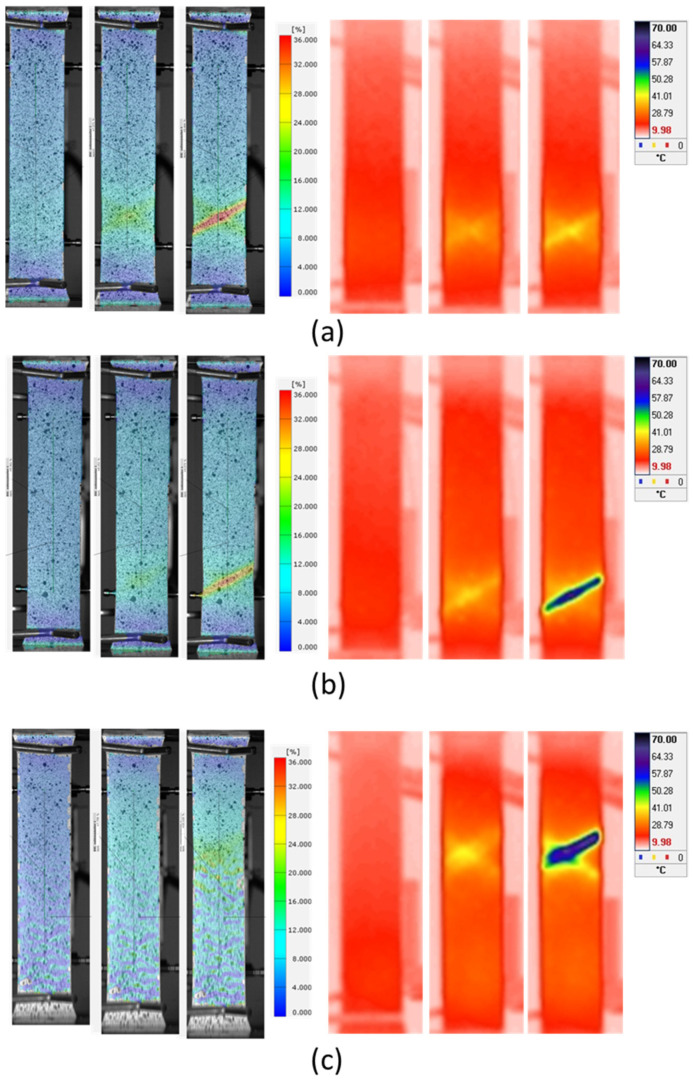
Von Mises equivalent strain distribution (2D DIC) and IR thermal distribution for specimen with orientation of 0° during uniaxial tensile tests conducted at velocities of (**a**) 10 mm/s, (**b**) 50 mm/s, and (**c**) 284 mm/s.

The experimental results show the effectiveness of digital image correlation (DIC) in capturing deformation across specimen surfaces. While the mechanical extensometer and DIC had the highest number of readings for the quasi-static test at 0.01 mm/s velocity, the IR camera was not able to capture temperature changes. The IR method is not suitable for low-rate loading velocities due to the high thermal conductivity of the copper alloy sheet and heat convection causing dissipation of the thermal effect. Samples subjected to higher strain rates have been successfully acquired with both methods, IR and DIC, so the results can be compared. Figure 8 presents the Von Mises equivalent strain distribution and thermal distribution for specimens oriented at 0° at loading velocities of 10 mm/s, 50 mm/s, and 284 mm/s. Each set of three images (DIC and IR) represents different stages of deformation: initial, mid-stage, and near fracture. The provided figures corresponding to different deformation stages enable the analysis of strain and thermal evolution during the tensile test. From these figures, it can be seen that as the tensile specimen is loaded, temperature and strain increase along the whole surface. When comparing IR thermography and the DIC method, one can observe an increase in temperature and strain occurring at the same location on the specimen. This correlation provides evidence that the generated heat is indeed a consequence of plastic deformation. Prior to a specimen failure, strain and temperature localize and form an angled slide line (in the form of an “X”), which can be seen in Figure 8.

**Figure 9:**
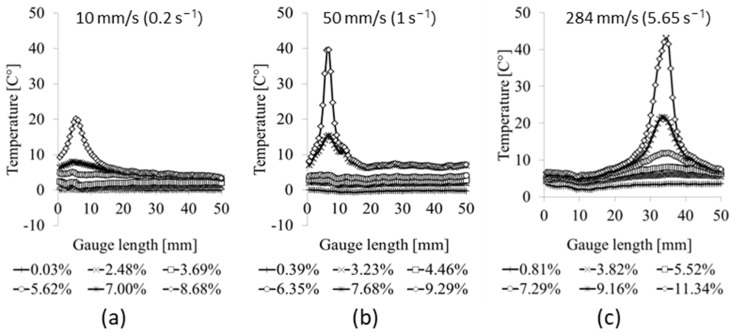
Temperature rise distribution of the specimen surface along longitudinal axis 1 for selected specimen with orientation of 0° at loading velocities of (**a**) 10 mm/s, (**b**) 50 mm/s, and (**c**) 284 mm/s.

The experimental investigation of the inelastic heat fraction (IHF) *β* across tensile specimens with different alignments to the rolling direction in the tests performed at different strain rates provided insight into the material’s thermomechanical behavior. As previously mentioned, the results obtained for a strain rate of 0.0002 s^−1^ were not analyzed due to heat dissipation at lower tensile velocities due to the pronounced effect of heat dissipation, which could affect the calculation of the inelastic heat fraction. For the selected specimen with an orientation of 0°, surface temperature rise distributions along the 50 mm gauge length at different elongations are presented in Figure 9. The gauge length is aligned with axis 1, representing the longitudinal direction of the specimen positioned in the same way as the DIC extensometer shown in Figure 4.

With the utilization of IR thermography, it is possible to distinguish between elastic and plastic deformation. For the uniaxial case, the first law of thermodynamics is given by the following relation:

(1) $$\rho c\dot{T}-k\frac{\sigma^{2}T}{\sigma_{x}^{2}}=\beta \sigma {\dot{\varepsilon }}^{p}-\alpha ET{\dot{\varepsilon }}^{e},$$

where *T* is the temperature, *σ* denotes stress, *σ_x_* denotes stress in the longitudinal direction, *ε^e^* is the elastic strain, *ε^p^* represents the plastic strain, *ρ* is the mass density, *c* is the specific heat capacity, *k* is the thermal conductivity, *α* is the thermal expansion coefficient, *E* is the elastic modulus, and *β* stands for the fraction of the plastic work converted into thermoplastic heating. Assuming adiabatic conditions and higher strain rates, with thermoelastic heating $\dot{Q^{e}}=\alpha ET{\dot{\varepsilon }}^{e}$ being small compared to thermoplastic heating, Equation (1) may be expressed in the following form:

(2) $$\rho c_{v}\dot{T}=\beta \sigma {\dot{\varepsilon }}^{p}.$$

Then, the inelastic heat fraction *β* can be defined as

(3) $$\beta =\frac{\rho c_{v}\dot{T}}{\sigma {\dot{\varepsilon }}^{p}},$$

where *c_v_* is the specific heat capacity at a constant volume, as the plastic flow is an isochoric process.

Based on the specimen surface temperature rise according to Figure 8 and Figure 9, mean temperature values were calculated for each tested sample as shown in Table 3. According to Equation (3), the calculated *β* coefficient exhibits differences for various orientations and various strain rates.

### 3.3. Breaking Angle

The fracture characteristics of tensile specimens were assessed using an optical measurement technique. The primary focus of this analysis was the determination of the breaking angle *ϴ*, a critical parameter for evaluating the fracture behavior of the material. The objective was to capture the breaking angle of the tensile specimens aligned differently to the rolling direction across various strain rates. The values of the measured averaged breaking angles are presented in Table 4. It can be observed that the strain rate does not affect the breaking angle *ϴ*. On the other hand, the breaking angle is significantly influenced by specimen orientation.

**Figure 10:**
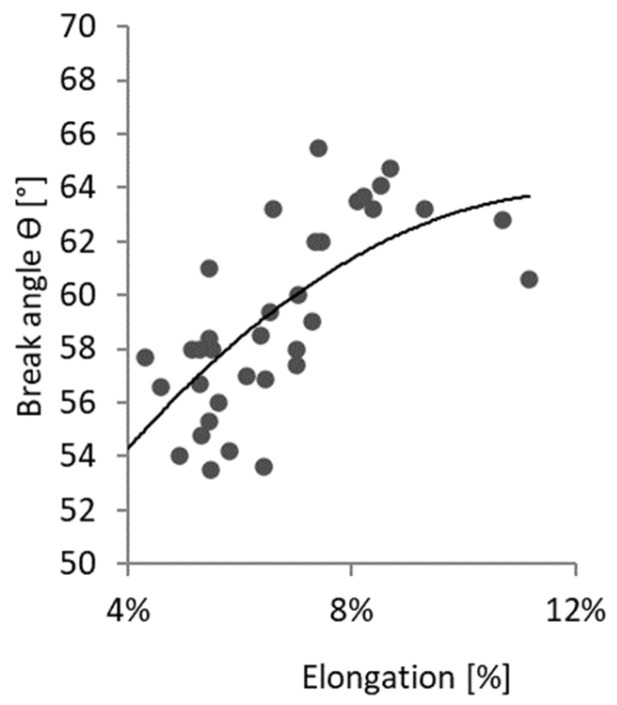
Specimen break angle in correlation with tensile elongation at break for all samples at all strain rates.

In Figure 10, values of the breaking angle corresponding to tensile elongation at break are presented for all tested samples and all considered strain rates. By considering these results, an unexpected trend can be noticed: with the increase in elongation, the breaking angle also increases.

**Figure 11:**
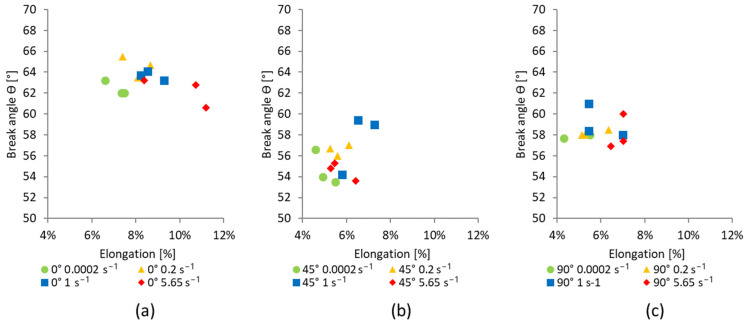
Strain rate influence on the breaking angle of specimens with alignment to the rolling direction: (**a**) 0°, (**b**) 45°, and (**c**) 90°.

Figure 11 presents the influence of the tensile specimen orientation and strain rate on the breaking angle for the considered CuFe2P sheet material. The results are presented for all tested samples, i.e., three samples for each considered orientation and each strain rate. The breaking angle is a critical parameter in metal forming as it reflects the slope at which a metal begins to fracture or fails under stress.

### 3.4. Lankford Coefficient

The Lankford coefficient, or *r*-value, is defined as the ratio of the true plastic strain in the width direction $\epsilon_{22}^{p}$ to the true plastic strain in the thickness direction $\epsilon_{33}^{p}\;$during uniaxial tensile testing, according to [36]:

(4) $$r_{\theta }=\frac{\epsilon_{22}^{p}}{\epsilon_{33}^{p}}=\frac{ln\left(\frac{w}{w_{0}}\right)}{ln\left(\frac{t}{t_{0}}\right)}.$$

Due to the assumption of the incompressibility hypothesis, the equation for thickness strain can be expressed as

(5) $$\epsilon_{33}^{p}=-(\epsilon_{22}^{p}+\epsilon_{11}^{p})$$

and the Lankford coefficient can be calculated as

(6) $$r_{\theta }=\frac{\epsilon_{22}^{p}}{-(\epsilon_{22}^{p}+\epsilon_{11}^{p})}=\frac{ln\left(\frac{w}{w_{0}}\right)}{-\left(ln\left(\frac{w}{w_{0}}\right)+ln\left(\frac{l}{l_{0}}\right)\right)}.$$

In the above equations, *w*, *t*, and *l* are the width, thickness, and length, respectively, in combination with subscript 0, which represents their initial values.

**Figure 12:**
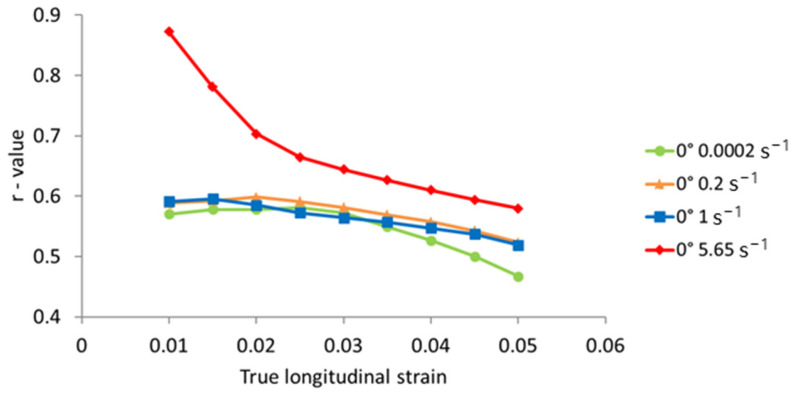
Lankford coefficient at different longitudinal true strains for specimens with orientation of 0°.

In order to obtain the Lankford coefficient for the tested orientations, the digital image correlation (DIC) method was utilized. Based on the measurements, total strains in the specimen’s width and longitudinal directions are obtained for the defined area of the specimen. The Lankford coefficient is then calculated as the ratio of the width and thickness plastic strain measures, according to Equation (6). For the specimens with an orientation of 0°, under different strain rate conditions, the Lankford coefficient is calculated for different values of the longitudinal true strain. The results are presented in Figure 12. It can be observed that the *r*-value changes with the ongoing deformation process during tensile testing.

**Figure 13:**
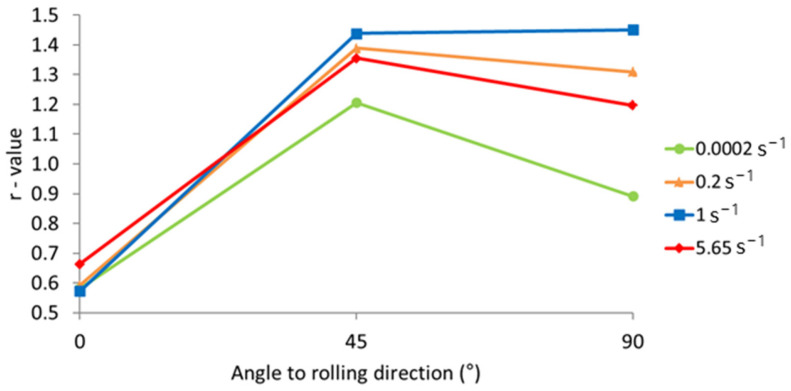
Influence of the strain rate on orientational dependence of Lankford coefficient calculated at true longitudinal strain corresponding to 0.025.

In Figure 13, the influence of the strain rate on the orientational dependence of the Lankford coefficient, calculated at 2.5% of the true longitudinal strain, is presented. Since, for different orientations, *r*-values are significantly different, it can be stated that the material exhibits highly anisotropic behavior.

## 4. Discussion

For a CuFe2P alloy sheet with a 0.8 mm thickness, uniaxial tensile tests were performed on specimens with orientations of 0°, 45°, and 90° relative to the rolling direction. The tests are performed at different strain rates, ranging from a value of 0.0002 s^−1^, corresponding to the quasi-static test, to higher values of 0.2 s^−1^, 1 s^−1^, and 5.65 s^−1^. The influence of specimen orientation and strain rate on the uniaxial tensile mechanical properties was considered.

As evidenced by the experimental results, the tensile strength is significantly influenced by both the specimen orientation and the strain rate. For all the strain rates considered, the highest average tensile strength values were obtained for specimens with an orientation of 90°, while the lowest values were obtained for specimens with an orientation of 45° (Figure 7c). For the quasi-static test, the average tensile strength amounted to 542 MPa, 515 MPa, and 547 MPa for orientations of 0°, 45°, and 90°, respectively (Figure 7c). Tensile strength increased with strain rate for all specimen orientations. For the considered strain rate range, the increase in average tensile strength amounted to 7%, 5.8%, and 7.7% for orientations of 0°, 45°, and 90°, respectively. The greatest tensile strength increase for all orientations was observed for the transition from the quasi-static test to the first higher strain rate considered, which amounted to approximately 5%.

The elastic modulus depends on the specimen orientation while showing minor variation as the strain rate increases. Similar to the tensile strength, the highest values of the elastic modulus were obtained for specimens with an orientation of 90°, while the lowest were obtained for specimens with an orientation of 45° (Figure 7a). The average values of the elastic modulus amounted to 128 GPa, 105 GPa, and 132 GPa for orientations of 0°, 45°, and 90°, respectively.

The amount of elongation strongly depended on the specimen orientation and the strain rate (Figure 7b). Specimens with an orientation of 0° showed the highest elongation, while those with an orientation of 90° generally exhibited the lowest values. For the quasi-static test, the average elongation for orientations of 0°, 45°, and 90° amounted to 7.1%, 5%, and 4.1%, respectively. For the considered strain rate range, for orientations of 0° and 90°, average elongation distributions increased monotonically, resulting in corresponding increases of 41.4% and 66.7%. For an orientation of 45°, the average elongation distribution had a maximum value for the strain rate value of 1 s^−1^, with a deviation from the quasi-static test of 31%. This suggests that material ductility increased at higher strain rates, which could have implications for forming processes where material ductility is a critical factor. The observed elongation increase for the strain rate increase contrasts with the reported results of similar studies found in the literature. Elongation decreasing with an increase in strain rate is usually reported for metals, for instance, in studies incorporating aluminum alloy metal sheets AA6019-T4, AA6061-T4, and AA2139-T351 [37,38]. However, a study of the influence of strain rate on uniaxial tensile properties for nano- and ultrafine-grained (UFG) copper showed that for 200 nm UFG copper, elongation increased with strain rate, while for 110 nm UFG copper, strain rate did not influence elongation [24]. Furthermore, the same study reported that for nano-grained copper with a grain size of 59 nm, elongation decreased with an increase in strain rate. These observations indicate the influence of grain size on macroscopic material behavior under different loading conditions. Furthermore, an increase in elongation with an increase in strain rate is reported for the AA6016 alloy sheet in different tempers [20].

As can be seen from Figure 11, the breaking angle is orientation-dependent, while the strain rate has minimal impact on its values. Specimens oriented along the rolling direction exhibited the highest values of breaking angle, ranging between 60.6° and 65.5°. Specimens oriented at 45° to the rolling direction had the lowest values, ranging between 53.5° and 59.4°, while specimens perpendicular to the rolling direction had values ranging between 56.8° and 61°. This difference arises as a result of the manufacturing rolling process, which leads to changes in the material microstructure. The dependency of the breaking angle on specimen orientation highlights the importance of considering manufacturing-induced anisotropy when predicting the fracture behavior of materials in real-world applications.

Using the DIC method and IR thermography, the determination of the inelastic heat fraction was feasible by analyzing strain and heat generation on the specimen surface. The results showed that the inelastic heat fraction (IHF) was dependent on strain rate and specimen orientation. According to the results presented in Table 3, the specimen with the lowest IHF of 0.51 had an orientation of 0° at a strain rate of 0.2 s^−1^, and the specimen with the highest IHF of 0.88 had an orientation of 45° at a strain rate of 5.65 s^−1^. For the considered strain range, the increase in average inelastic heat fraction amounted to 41.2%, 43.7%, and 15.7% for orientations of 0°, 45°, and 90°, respectively. For all specimen orientations, the highest values were achieved at the highest strain rate. The results indicate that the IHF coefficient is not a constant value, as traditionally assumed and widely used in the literature [28]. This conclusion has also been addressed by other authors [29,30]. The non-linear evolution of IHF, especially pronounced in specimens tested at higher strain rates, implies that the material’s ability to convert plastic work into heat changes as deformation progresses. These experimental results show the importance of considering the strain rate and material anisotropy when evaluating the amount of work converted into heat. The observed dependency of IHF on these factors shows the need for more sophisticated models that can accurately predict energetic processes occurring during plastic deformation.

Figure 12 and Figure 13 show that the Lankford coefficient (*r*-value) depends on both the specimen orientation and the strain rate. For the quasi-static test, the *r*-values amounted to 0.58, 1.21, and 0.89 for orientations of 0°, 45°, and 90°, respectively. For the selected specimen with an orientation of 0°, the *r*-values calculated at selected values of true longitudinal strain showed a decrease with the ongoing deformation process in the tensile test. This alternation was the most pronounced for the highest strain rate considered, where a rapid reduction in the *r*-value with longitudinal strain was observed (a reduction of 34% for the longitudinal strain range Δ = 0.04). For lower strain rates, the alternation of the *r*-value with the ongoing deformation was less pronounced (a reduction of approximately 18% for the lowest strain rate). These results clearly indicate that strain rate has an impact on *r*-values. Furthermore, strain rate had the smallest impact on *r*-values for an orientation of 0° and the highest for an orientation of 90°. There is no clear indication that higher strain rates will result in greater *r*-values for all specimen orientations, but there is a trend of *r*-value increase between the quasi-static test and higher strain rates. Traditionally, *r*-values are considered constant in anisotropic material models. However, the presented experimental data revealed that *r*-values vary with the ongoing deformation process and are affected by strain rate, challenging the assumption of constancy and emphasizing the importance of strain rate in assessing material anisotropy.

## 5. Conclusions

This study presents an investigation of the anisotropic elasto-plastic behavior of a CuFe2P sheet with 0.8 mm thickness using advanced experimental techniques, digital image correlation (DIC), and infrared (IR) thermography. Uniaxial tensile tests were conducted under different strain rates (0.0002 s^−1^, 0.2 s^−1^, 1 s^−1^, and 5.65 s^−1^) on sheet specimens with orientations of 0°, 45°, and 90° relative to the rolling direction. The primary focus was to understand how, for the considered material, strain rate affects uniaxial tensile mechanical properties. Based on the obtained experimental data, the following conclusions are derived:

- The material considered shows pronounced anisotropy in all considered uniaxial tensile mechanical properties, i.e., the stress–strain curve, the elastic modulus, the tensile strength, the elongation, and the Lankford coefficient, which significantly depend on the specimen orientation relative to the rolling direction.
- An increase in strain rate generally resulted in increased values of tensile strength and elongation for all tested orientations, while the elastic modulus remained almost constant.
- The values of the Lankford coefficient are influenced by the strain rate; the lowest values were obtained for the lowest strain rate considered. By calculating the values of the Lankford coefficient at different values of longitudinal plastic strain, it is observed that this coefficient decreases with the ongoing deformation process. This alternation is most pronounced for the highest strain rate considered. These observations challenge the conventional assumption of the Lankford coefficient being constant.
- The inelastic heat fraction (IHF) varied with strain rate and specimen orientation relative to the rolling direction, indicating that the material’s capacity to convert plastic work into heat depends on loading conditions. Generally, IHF increases with strain rate, with samples oriented at 45° to the rolling direction exhibiting the highest values.
- The breaking angles assessed in the tensile tests performed show that specimen orientation influences fracture behavior, while strain rate has a minor effect. The highest breaking angles were observed for specimens aligned with the rolling direction (0° orientation), while specimens oriented at 45° to the rolling direction exhibited the lowest breaking angles.

Further investigation will be focused on modeling the thermoplastic material behavior of the CuFe2P sheet material, aiming to accurately represent experimental findings through numerical simulations. An accurate constitutive model for the considered material should take into account material anisotropy, strain rate influence, and heat convection at non-adiabatic speeds. Moreover, incorporating microstructural analyses could clarify the underlying mechanisms influencing anisotropy and strain rate sensitivity.

## Figures and Tables

**Table 1:** Chemical composition (wt%) of CuFe2P.

Element	Fe	Zn	P	Cu
Composition (wt%)	2.1–2.6	0.05–0.2	0.015–0.15	rest

**Table 2:** Strain rates of uniaxial tensile tests.

Strain rate (s^−1^)	0.0002	0.2	1	5.65

**Table 3:** Inelastic heat fraction at different strain rates for selected specimens with orientations of 0°, 45°, and 90° relative to the rolling direction.

*β*			
	0.2 s^−1^ (10 mm/s)	1 s^−1^ (50 mm/s)	5.65 s^−1^ (284 mm/s)
0°	0.51 ± 0.07	0.66 ± 0.05	0.72 ± 0.04
45°	0.61 ± 0.05	0.79 ± 0.05	0.88 ± 0.07
90°	0.70 ± 0.01	0.72 ± 0.01	0.81 ± 0.06

**Table 4:** Average breaking angle at different strain rates for specimens with orientations of 0°, 45°, and 90° relative to the rolling direction.

*ϴ*				
	0.0002 s^−1^ (0.1 mm/s)	0.2 s^−1^ (10 mm/s)	1 s^−1^ (50 mm/s)	5.65 s^−1^ (284 mm/s)
0°	62.4° ± 0.4°	64.6° ± 1.1°	63.7° ± 0.5°	62.2° ± 1.6°
45°	54.7° ± 1.2°	56.6° ± 0.6°	57.5° ± 3.3°	54.6° ± 0.9°
90°	57.5° ± 0.7°	58.2° ± 0.2°	59.1° ± 1.1°	58.1° ± 1.2°

## Data Availability

The data presented in this study are available upon reasonable request from the corresponding author.
